# Are Dietary Patterns Related to Cognitive Performance in 7-Year-Old Children? Evidence from a Birth Cohort in Friuli Venezia Giulia, Italy

**DOI:** 10.3390/nu14194168

**Published:** 2022-10-07

**Authors:** Michela Marinoni, Elisa Giordani, Cedric Mosconi, Valentina Rosolen, Federica Concina, Federica Fiori, Claudia Carletti, Alessandra Knowles, Paola Pani, Maura Bin, Luca Ronfani, Monica Ferraroni, Fabio Barbone, Maria Parpinel, Valeria Edefonti

**Affiliations:** 1 Department of Medicine—DAME, University of Udine, Via Colugna 50, 33100 Udine, Italy; 2 Branch of Medical Statistics, Biometry, and Epidemiology “G.A. Maccacaro”, Department of Clinical Sciences and Community Health, Università degli Studi di Milano, Via Celoria 22, 20133 Milan, Italy; 3 Institute for Maternal and Child Health, IRCCS “Burlo Garofolo”, Via dell’Istria 65/1, 34137 Trieste, Italy; 4 Fondazione IRCCS Ca’ Granda Ospedale Maggiore Policlinico, Via Francesco Sforza 35, 20122 Milan, Italy; 5 Institute of Hygiene and Clinical Epidemiology, Azienda Sanitaria Universitaria Friuli Centrale, Via Colugna 50, 33100 Udine, Italy

**Keywords:** dietary record, dietary patterns, factor analysis, overall dietary exposure, nutrients, primary school children, cognitive performance, neurodevelopment, WISC-IV

## Abstract

Research from different sources supports a link between nutrition and neurodevelopment, but evidence is still sparse regarding the relationship between *a posteriori* dietary patterns (DPs) and cognitive performance in school-aged children. Within the Northern Adriatic Cohort II, Friuli Venezia Giulia, Italy, 379 7-year-old children were cross-sectionally evaluated. Dietary patterns were identified through a principal component factor analysis based on 37 nutrients from children’s 3-day dietary records. The Wechsler Intelligence Scale of Children (WISC-IV) test provided measures of cognitive performance, including the full-scale intelligence quotient (FSIQ) and single index scores. Each DP was related to FSIQ or single index scores through multiple robust linear regression models. We identified five DPs named “Dairy Products”, “Plant-based Foods”, “Fats”, “Meat and Potatoes”, and “Seafood” (63% of variance explained). After adjustment, no significant relationship was observed with the FSIQ score; positive associations were found between the “Seafood” DP and Verbal Comprehension Index or Perceptual Reasoning Index. The “Meat and Potatoes” and “Dairy Products” DPs were inversely associated with the Verbal Comprehension Index and Processing Speed Index scores, respectively. In the absence of a relation with the overall FSIQ score, single DPs might influence specific cognitive functions, including verbal and reasoning abilities, as targeted by single indexes, in the expected direction.

## 1. Introduction

Cognition is a complex set of brain functions that support information learning, storage, retrieval, and processing [1]. Those abilities have been reported to have a major role in predicting school achievement [2].

A large body of research supports links between nutrition and neurodevelopment [3,4,5]. Traditional approaches consider the effect of single-nutrient supplementation on neurodevelopment [6], following the rationale emerged from mechanistic studies linking inadequate intakes to specific neurodevelopmental processes [3,4]; results of supplementation were more evident in micronutrient-deficient children than in healthy subjects [6]. Similarly, extended literature has considered single nutrients/foods consumed by children and adolescents in real life [5], but it still focuses on linking inadequate consumption, including fish (with corresponding low intakes of polyunsaturated fatty acids (PUFAs), especially docosahexaenoic acid (DHA) and eicosapentaenoic acid (EPA)), fruit and vegetables (with corresponding low intakes of flavonoids), and/or fast-food products (with high intakes of saturated fats and refined carbohydrates), to poorer cognitive functions and academic achievement. 

Whilst the roles of individual nutrients or foods have been investigated, interactions among and between macronutrients and micronutrients deserve further investigation with regards to their possible effects on brain development in children. In addition, the traditional approach to the diet—neurodevelopment association does not reflect the real-life scenario of food consumption, where free-living individuals do not eat isolated foods and, within foods, nutrients have well-known synergistic effects [7]. Similarly, possible interactions between nutrients and contaminants might be of interest in a comprehensive evaluation of the role of dietary exposure in neurodevelopment [8].

Diet complexity can be better disentangled using dietary patterns (DPs). *A posteriori* dietary patterns [9], defined by using multivariate statistics (i.e., principal component analysis (PCA), factor analysis (FA), and cluster analysis) [10], are advantageous in naturally capturing actual dietary behavior and its possible determinants (e.g., geography/climate, socioeconomic status, food supply, ethnic background, religion), compared to the *a priori* (i.e., comparing subjects’ diet against evidence-based benchmark diets) options. When derived on dietary records, the *a posteriori* DPs provide a comprehensive but more detailed picture of dietary habits, to be linked to specific brain maturation processes. Among the *a posteriori* DPs, FA-based DPs provide a straightforward representation of the actual dietary profiles through the factor-loading matrix. For this reason, they are usually preferred to those from a cluster analysis [11].

A few previous papers have related *a priori* [12,13,14,15,16,17] and *a posteriori* [18,19,20,21,22,23,24,25] DPs to cognitive/academic performance in cross-sectional and/or longitudinal analyses. Cross-sectional analyses of *a priori* DPs, including the Mediterranean Diet score [12,13,14,17], the Healthy Eating Index [15], the Dietary Approach to Stop Hypertension (DASH) diet score [16], the Finnish Children Healthy Eating Index [16,17], and the Baltic Sea Diet Score [16,17], suggested that higher diet quality indices are generally associated with better cognitive and academic performance in children and adolescents from Europe and the United States. Cross-sectional and longitudinal analyses generally revealed that unhealthy *a posteriori* DPs, characterized by energy-dense and processed foods as well as refined grains, are related to lower cognitive/academic performance [18,19,20,21,22,23,24,25].

The Northern Adriatic Cohort II (NAC-II) is a birth cohort from the Friuli Venezia Giulia region in the north east of Italy, originally established to investigate the association between prenatal mercury (Hg) exposure from maternal fish consumption and child neurodevelopment at 18 months of age [26,27]. Follow-up information from this cohort provides the unique opportunity to track further key neurodevelopment stages and to improve our understanding of the role of diet on cognition in Italian 7-year-old children. 

This paper tackles diet complexity with the use of *a posteriori* DPs from FA and relates it with the complexity of the brain’s neurodevelopmental processes, as measured by the Wechsler Intelligence Scale for Children–Fourth Edition (WISC-IV) in 7-year-old children.

## 2. Materials and Methods

### 2.1. Design and Participants

Dietary habits and cognitive performance of Italian 7-year-old children were collected in a cross-sectional analysis nested within the NAC-II study [28]. Briefly, the NAC-II enrolled 900 pregnant women from Friuli Venezia Giulia region, Italy, between 2007 and 2009 to investigate the association between Hg exposure from maternal fish consumption during pregnancy and child neurodevelopment (for a detailed description of the study protocol, see [26,27]). The corresponding 767 eligible children born from 767 mothers were followed-up with: (i) supplementary questionnaires administered at 18 months, 40 months, and 7 years, and (ii) assessment of the children’s neurodevelopment at different ages (for a detailed description of the entire data collection process, see [28]).

The study was conducted according to the Declaration of Helsinki and caregivers provided informed consent. The research protocol was approved by the Ethics Committees of the University of Udine and Institute for Maternal and Child Health IRCCS Burlo Garofolo.

### 2.2. Cognitive Performance

Cognitive abilities were assessed by trained psychologists for 487 out of 632 eligible (i.e., children with neurodevelopment assessed at 18 months) 7-year-old children. 485 of them were administered the Italian version of the WISC-IV [29,30]. Details on the validation process of the Italian version of WISC-IV has been provided elsewhere [30]. The WISC-IV assessed children’s general cognitive ability as a full-scale intelligence quotient (FSIQ) [29,31]. In addition, four more indexes targeted verbal comprehension (VCI), perceptual reasoning (PRI), working memory (WMI), and processing speed (PSI). The FSIQ and single indexes were standardized to have a mean of 100 and a standard deviation of 15 by using the Italian population-based reference data (standard population), and they range from 40 to 160. Higher values of the FSIQ or the single indexes represent a better test performance and therefore stronger cognitive abilities. A total of 481 children provided scores on each index and therefore on the FSIQ score; four children lacked information on the VCI score and therefore on the FSIQ score.

### 2.3. Dietary Assessment

Within the overall sample of 485 children with at least one WISC-IV index score at 7 years, 379 caregivers filled in a three-day-dietary record (3-dDR) to assess their child’s diet (2 weekdays and 1 weekend day, not necessarily consecutive) in the week before the neurodevelopmental assessment, over a total of 381 caregivers who originally provided the 3-dDR. In the final sample of 379 caregiver/child pairs, information on diet and cognitive performance was complete for 376 of them, whereas 3 children lacked information on the VCI score and therefore on the FSIQ score.

Nutrient and energy intakes were derived from the collected food items using the Microdiet Software V4.4.1 (Microdiet software-Downlee Systems Ltd., High Peak, Salford, UK), which contains the “Food Composition Database for Epidemiological Studies in Italy” [32]. When available, nutritional label data, standard recipes, and portion sizes were used in the calculation of nutrient intakes. Information on food fortification or supplement use was not collected within the 3-dDR [33]. The 828 available single food items from the 3-dDRs were grouped into 35 food groups. For every child, the food group intake was expressed on a daily basis by dividing it for the total number of recording days. The full list of food groups and the corresponding food items are presented in Supplementary Table S1. 

### 2.4. Socio-Demographic, Socio-Economic, and Lifestyle Characteristics

Among other procedures, information collected during pregnancy included mother’s smoking intensity and status, alcohol consumption, pre-pregnancy weight, and maternal IQ assessed using the Raven’s Progressive Matrices Test (Raven’s test score) [34]. The socio-demographic and socio-economic characteristics of both parents, including education level, marital status, citizenship, and house property and size, were obtained from the questionnaire administered one month after delivery. Between 18 and 40 months of age of the child, an interview with the caregivers and an observation of parent–child interaction in the home context by trained psychologists provided the basis for the assessment of the AIRE instrument, designed and validated by Capotorti et al. [35] to adapt the Home Observation for Measurement of the Environment (HOME) model [36] to conform with the Italian culture and social organization [37].

The self-administered Recall Follow-up Questionnaire at 7 years of age of the child was mainly focused on children’s lifestyle habits, including physical activity, sedentary behaviors, and passive smoking exposure. Children’s height and weight were also measured by healthcare staff [28]. Body mass index (BMI; kg/m^2^) was calculated as: [weight (kg)/height^2^(m^2^)]. Children were categorized as underweight, normal weight, overweight, or obese, according to the World Obesity Federation [38] based on International Obesity Task Force (IOTF) BMI cut-offs [39]. The lifestyle habits of parents were also updated, including smoking and alcohol consumption.

### 2.5. Statistical Analysis

#### 2.5.1. Selecting Nutrients of Interest

From the “Food Composition Database for Epidemiological Studies in Italy” [32] list of 96 available nutrients, we selected 37 that best represented the overall diet of 7-year-old Italian children. In addition to all the caloric components, we expanded as much as possible the fatty acid profile and included minerals and vitamins previously related to cognitive performance [5]. 

#### 2.5.2. Checking Factorability

We integrated visual inspection with quantitative measures, such as Bartlett’s test of sphericity and overall (Kaiser–Meyer–Olkin) and individual measures of sampling adequacy [40], to evaluate the factorability of the correlation matrix of the original nutrients.

#### 2.5.3. Identifying Nutrient-Based Dietary Patterns

After reassuring factorability checks, *a posteriori* DPs of 7-year-old children were identified through an exploratory principal component factor analysis (PCFA) on the correlation matrix of the 37 selected nutrients. PCFA describes the correlation structure of nutrients in terms of a smaller number of underlying unobservable and randomly varying factors, known as DPs from now on [41]. We selected the number of factors to retain, taking advantage of the following criteria: factor eigenvalue > 1, scree-plot visual inspection, and factor interpretability [41]. We applied a varimax rotation to obtain a simpler loading structure with a better interpretation. We adopted a quantitative criterion to name DPs, where “dominant nutrients” are those showing rotated factor loadings ≥ |0.60|. Indeed, a 0.60 cut-off implies a minimum contribution of any DP to any nutrient’s total variance of more than 35% (i.e., 0.60^2^ = 0.36) [42]. Children’s factor scores were estimated for each DP using the weighted least squares method. They indicate to what extent each individual’s diet was adherent to each DP [40].

#### 2.5.4. Assessing Internal Reproducibility, Internal Consistency, and Validity of the Identified Dietary Patterns

To evaluate the internal reproducibility of the identified DPs, we performed additional analyses using different estimation methods (i.e., principal axis factor analysis (PAF) with the generalized least squares estimation method and maximum likelihood factor analysis (MLFA), after logarithmic transformation of the original nutrients), a different orthogonal rotation method (i.e., quartimax), and a different procedure for estimating factor scores (i.e., multiple regression method) [41]. Congruence coefficients (CCs) between factor loadings from apparently similar DPs were used for the evaluation of internal reproducibility according to the following cut-offs: 0.85 ≥ CC ≤ 0.94 indicates “fair similarity” and a CC > 0.95 “equivalence” of the identified DPs [43]. Since results were consistent, we performed all the subsequent analyses on factor scores obtained from the main analysis based on PCFA, with varimax rotation and the weighted least squares method. We also assessed the internal consistency of the identified DPs using standardized Cronbach’s alphas and alphas when-item-deleted (nutrient loadings ≥ |0.40| only) [44]. To further validate the identified DP, we calculated the Pearson correlation coefficients between the PCFA-based continuous factor scores and the intake (g/day) of the 35 selected food groups [45,46,47] (see Supplementary Table S1 for a detailed description of the food group list).

#### 2.5.5. Relating Identified Dietary Patterns with Cognitive Performance

The association between DPs and WISC-IV FSIQ and index scores was assessed using linear regression models. The dependent variable, FSIQ (or index) score (continuous variable), was regressed against the DP scores (continuous variables, main independent variables, uncorrelated due to PCFA and varimax rotation), controlling for observable confounding factors (additional independent variables of mixed type). Violations of the standard ordinary least squares assumptions suggested that we use the robust MM estimator for our analysis [48].

For each dependent variable, selection of the final model, including the best set of confounders, started from the composite model including all the five factors simultaneously and added confounders one at a time based on the likelihood ratio test of significance. Variables were examined in the following groups: infant characteristics (i.e., sex, birth weight, breastfeeding (yes vs. no and duration in months)), maternal and paternal characteristics (i.e., maternal age, education of both parents, maternal age at delivery, folic acid supplementation before and during pregnancy, home size, house property, AIRE scores (4 subscales, 0–20 each) together with age at test administration, and, for the mother only, alcohol, smoking, fish consumption during pregnancy, and Raven’s test score), child characteristics at 7 years of age (passive smoking, BMI, physical activity, use of TV during weekdays and weekends, use of any device (including video-games, computer, tablet, and smartphone) during weekdays and weekends). To improve the comparability of the results, we used the same list of confounding factors in all the final models, independently of the dependent variable. This final list included the following variables: father’s education, maternal Raven’s test score during pregnancy, folic acid supplementation before pregnancy, alcohol consumption during pregnancy, breastfeeding, house property, child’s sex, child’s birth weight, child’s BMI at 7 years, and child’s extracurricular physical activity at 7 years (see Table 1 for categories of confounding factors).

Two-way interactions between each DP and selected confounding factors were added to the final model one at a time, giving a total of 250 additional interaction-term models fitted (5 outcomes times 5 factors times 10 confounders). The significance of the interaction terms was assessed with the likelihood ratio test between the final model and each interaction-term model. Adjusted parameter estimates (beta coefficients), standard errors, and *p*-values from the corresponding hypothesis test were provided for each final model. 

A sensitivity analysis was carried out to further adjust the final model for AIRE scores (together with age at test administration), use of TV during weekdays and weekends, and use of devices during weekdays and weekends, which were potentially relevant confounding factors but showed a high number of missing values (*N* = 48, 5, and 60, respectively). An additional sensitivity analysis excluded preterm births (i.e., gestational age < 37 weeks, *N* = 5) from each final model (see Supplementary Table S2 for categories of additional confounding factors and corresponding frequencies).

Calculations were carried out using the open-source statistical computing environment R [49,50], with its libraries “psych” [51] and “GPArotation” [52] for the identification of DPs and “MASS” [53] for robust regression.

## 3. Results

### 3.1. Population Characteristics

Socio-demographic, socio-economic, socio-environmental, and lifestyle characteristics of the examined sample are provided in Table 1 and Supplementary Table S2 as mean ± standard deviation (SD), median, and first and third quartile for continuous variables and as frequency and percentage for categorical variables. The mean age of the 379 Italian children who participated in the present follow-up was 7 ± 0.05 years; percentages of females and males were comparable. Of them, approximately 67.3% were normal-weight subjects, whereas 17.7% and 4.7% were overweight and obese, respectively. A total of 43.3% of the children regularly practiced at least 1 h/day of extra-curricular physical activities 3 or more times a week; about 18% and 67% used any electronic device and watched TV more than 1 h/day during weekdays, respectively. During the weekend, percentages of children playing videogames and watching TV generally increased compared to weekdays. The mean and SD of the maternal Raven’s test score measured during pregnancy was 119 ± 11. About 42% of the mothers had already started folic acid supplementation before becoming pregnant, and almost 90% had breastfed their child. About 70% of the fathers had a high school diploma and 22.0% had a higher educational level; and the majority of parents were homeowners (~80%).

### 3.2. Cognitive Performance on the Wechsler Intelligence Scale for Children–Fourth Edition (WISC-IV)

Mean (SD), median (1st and 3rd quartile), and minimum and maximum values for FSIQ and index scores are presented in Table 2. In particular, the mean ± SD were 109 ± 11 for FSIQ, 108 ± 11 for VCI, 114 ± 11 for PRI, 98 ± 10 for WMI, and 102 ± 13 for PSI. All means fell within or exceeded the normal range (i.e., “average category”: 90–109) [54,55]. The first quartile reached the normal range for FSIQ and single index scores, suggesting that, for each score, 75% of the children had a normal or a higher-than-normal score [54,55].

### 3.3. Identification of Nutrient-Based Dietary Patterns

The factorability of the correlation matrix was reassuring, with the overall measure of sampling adequacy being equal to 0.84 and 33 out of 37 nutrient-specific measures of sampling adequacy exceeding 0.70 (Supplementary Table S3).

Table 3 gives the factor-loading matrix for the five retained factors, hereafter named as DPs, and the corresponding communalities. 

The identified DPs explained an overall ~63% of total variance, with single percentages ranging from 9% to 15%. The greater the nutrient loading to a factor (in absolute value), the higher the contribution was of that nutrient to that factor. Thus, the first pattern named “Dairy Products” (~15%) had the greatest loadings on calcium, biotin, magnesium, pantothenic acid, iodine, phosphorus, and vitamin B2. The second pattern named “Plant-based Foods” (~14%) had the greatest loadings on total fiber, vitamin C, folate, potassium, beta-carotene, vitamin E, and iron. The third pattern named “Fats” (~13%) had the greatest loadings on monounsaturated fatty acids (MUFAs), oleic acid, saturated fatty acids (SFAs), and linoleic acid. The fourth pattern named “Meat and Potatoes” (~12%) had the greatest loadings on niacin, vitamin B6, proteins, vitamin B1, and zinc. The fifth pattern named “Seafood” (~9%) had the greatest loadings on EPA, DHA, and selenium.

### 3.4. Internal Consistency and Reproducibility of the Identified Dietary Patterns

Nutrient communalities (Table 3) and the internal consistency of the identified DPs, as measured by Cronbach’s alphas (Supplementary Table S4), were satisfactory, further supporting the choice of the included nutrients. Indeed, communalities ranged between 0.32 and 0.87, with 25 nutrients exceeding 0.50. In addition, Cronbach’s coefficient alphas were around 0.90, except for the “Seafood” DP, where the alpha was equal to 0.75. Most alpha-when-item-deleted coefficients were smaller than the corresponding overall alpha coefficient for the same DP, but differences were generally negligible; for the dominant nutrients in the “Seafood” DP, decreases in the alpha-when-item-deleted were more pronounced but still within 20%.

The internal reproducibility of the derived DPs—as based on the comparison with MLFA and PAF—was good. In detail, the PAF-based DPs were all equivalent (CCs ≥ 0.99) to their counterparts from PCFA. Results from “Plant-based Foods”, “Dairy Products”, and “Fats” DPs were equivalent in MLFA and PCFA (all CCs ≥ 0.95). The reproducibility of the MLFA-based “Seafood” and “Meat and Potatoes” DPs was close to reach “fair similarity” (CC = 0.82 and =0.84, respectively) with PCFA-based ones. However, the MLFA-based “Seafood” DP shifted towards a more evident meat orientation: as compared to the PCFA-based “Seafood” DP; in the MLFA-based “Seafood” DP, EPA and DHA showed lower loadings (EPA: in MLFA 0.49 vs. 0.83 in PCFA; DHA: in MLFA 0.42 vs. 0.68 in PCFA), whereas arachidonic acid and niacin showed higher loadings (arachidonic acid: in MLFA 0.66 vs. 0.38 in PCFA; niacin: in MLFA 0.67 vs. 0.22 in PCFA). In addition, the MLFA-based “Meat and Potatoes” DP shifted towards a starch-based only DP, with starch reaching 0.70 in MLFA vs. 0.42 in PCFA.

### 3.5. Food Groups and Identified Dietary Patterns

Summary statistics of the distribution of each DP are provided in Supplementary Table S5. Table 4 gives the Pearson correlation coefficients between the identified DPs and the 35 selected food groups as captured on the same subjects. Median and 1st–3rd quartiles of daily intakes and the percentage of non-consumers are presented in Supplementary Table S1 for each food group.

The “Dairy Products” DP score was positively correlated with milk, yogurt, fat cheese, and bananas. The “Plant-based Foods” DP score had positive correlation coefficients with other fruits, coloured vegetables, bananas, green leafy vegetables, whole grains and whole bread, citrus fruits, fruit juices, milk substitutes, pulses and pulses products, other vegetables, and olive oil and olives. The “Fats” DP showed a positive correlation with olive oil and olives, processed and ultra-processed meat, fat cheese, refined grains, white bread and bread substitutes, spoon desserts, and chocolate; a negative correlation was observed for bananas. The “Meat and Potatoes” DP was positively correlated with poultry, processed and ultra-processed meat, red meat, potatoes, refined grains, white bread and bread substitutes, and breakfast cereals. The “Seafood” DP had positive correlation coefficients with fatty fish, crustaceans and shellfish, lean fish, and canned fish and was negatively correlated with ready-to-eat meals.

### 3.6. Cognitive Performance and Identified Dietary Patterns

In the absence of significant interactions (*p*-value < 0.001 from the likelihood ratio test on the interaction terms between each DP and each confounding factor) in most (97.5%) of the investigated models, Table 5 presents results on the relationship between the five identified DPs and cognitive performance (FSIQ and index scores), after adjustment for selected confounding factors within the main-effect model. For the same range (40–160) in FSIQ and index scores, the mean scores for the children in the reference category (males, normal weight status, 2 or less days/week of extracurricular physical activity at 7 years, birth weight < 4 kg, no breastfeeding, no folic acid supplementation before pregnancy, lowest father’s education level, and absence of house property) varied from ~69 (WMI) to 85 (PRI), with the FSIQ mean score being equal to ~72 (testing the null hypothesis of model intercept (i.e., mean score) being equal to 0: *p*-value< 0.001). None of the identified DPs were significantly related to the FSIQ score. However, three (out of four) WISC-IV-related single index scores were related to three (out of five) DPs. In detail, the VCI score depended on the “Seafood” and “Meat and Potatoes” DPs, with the “Seafood” DP exerting a direct effect (beta coefficient = 1.24, *p*-value < 0.05) and the “Meat and Potatoes” DP an inverse one (beta coefficient = −1.28, *p*-value < 0.05). The PRI score directly depended on the “Seafood” DP, with the beta coefficient estimate being equal to 1.35 (*p*-value < 0.05). The PSI score inversely depended on the “Dairy Products” DP, with the beta coefficient estimate being equal to −2.05 (*p*-value < 0.01). None of the identified DPs were significantly related to the WMI score. Additional contributions of selected confounding factors were in the expected direction. Major roles were played by mother’s Raven’s test score and father’s education, which were significantly and directly related to FSIQ and most of the index scores, except for PSI index. In addition, supplementation with folic acid before pregnancy was directly related to the FSIQ score and index scores (VCI and WMI).

When a significant interaction was detected (six combinations of DP and confounding factor out of 250 possible combinations), the number of subjects within each stratum was too small to provide reliable effect estimates.

The four sensitivity analyses provided reassuring results (Supplementary Tables S6–S9). After adjustment for the use of device (weekdays and weekend) (two variables, one model), TV watching (weekdays and weekend) (two variables, another model), and AIRE scores (four variables and age at test administration, another model), point estimates of beta coefficients and corresponding statistical tests were generally in line with the ones from the main analysis. In addition, the “Meat and Potatoes” DP reached significance in the model with the FSIQ score after adjustment for device use (beta coefficient = −1.26, *p*-value < 0.05); similarly, the beta coefficient was equal to −1.59, *p*-value < 0.01, for the same DP, when PRI score and use of device were considered. The “Fats” DP was significantly and inversely associated with VCI (beta coefficient = −1.20, *p*-value < 0.05, with adjustment for device, and beta coefficient = −1.03, *p*-value < 0.05, with adjustment for TV watching). When we restricted the analysis to full-term children (five pre-term children removed), beta coefficients for the “Seafood” DP reached 1.33 (*p*-value < 0.01) with VCI score and 1.47 (*p*-value < 0.01) with PRI score.

## 4. Discussion

We identified five major DPs among 7-year-old Italian children from the NAC-II cohort, named “Dairy Products”, “Plant-based Foods”, “Fats”, “Meat and Potatoes”, and “Seafood” DPs. These DPs explained a reasonably high (63%) proportion of the variability originally present in nutrient intakes of this sample. After adjustment for several selected confounders and the remaining DPs, no significant relationship was observed between each DP and the overall FSIQ score. In the analysis of single indexes, top-consumers of the “Seafood” DP had higher mean scores in verbal abilities and perceptual reasoning compared to those in the low-consumption category. In addition, top-consumers of the “Meat and Potatoes” DP showed a lower mean verbal ability score and those of the “Dairy Products” DP a lower mean processing speed score.

The comparison of results from studies assessing the association between *a posteriori* DPs and health/disease is always challenging, due to subjective decisions at various levels of the analysis. In a factor analysis, these include the type and number of dietary components to analyze, the preprocessing method, the number of factors to retain, the choice of applying a rotation or not (and which method to use), and the labelling of the identified factors [56]. When considering the relation between *a posteriori* DPs from PCA or FA and cognitive performance, additional challenges include the potentially different tools used for the assessment of diet and cognitive performance; in particular, the use of different psychometric tests across papers makes result comparisons difficult because each test evaluates cognitive performance through indexes that only partially overlap in terms of the explained underlying cognitive functions. In addition, within the few papers identified in the literature on this topic [18,19,20,21,25], target populations were substantially different and included adolescents from Malaysia [19] and China [25], 4-year-old children from Greece [18], and 7- and 8.5-year-old children from the UK-based Avon Longitudinal Study of Parents and Children (ALSPAC) cohort [20,21]. A detailed comparison of results across papers is therefore unfeasible.

The general picture that emerged from the literature points to weak associations between PCA- or FA-based DPs and overall measures of cognitive performance, when present [19,20,21], in cross-sectional analyses such as ours. One DP only was significantly associated with cognitive performance out of the three (“Processed”, “Traditional”, and “Health-conscious”) [21] or four (“Refined-grain”, “Snack-food”, “Plant-based food”, and “High-energy food”) [19] identified in the available studies. In the last paper [20], none of the three (“Junk food”, “Traditional”, and “Health-conscious”) DPs identified in 7-year-old children were significantly associated with the concurrent overall measure of school attainment. These results are in line with the lack of significant relationships between our DPs and the overall FSIQ score. One possible explanation for this substantial lack of statistically significant relationships considers the cross-sectional nature of the analyses. Diet might exert its detrimental or beneficial effects over a longer time span; therefore, integrating a longitudinal and a cross-sectional analysis within a longitudinal design might identify diet contribution at either time span. Results from a longitudinal analysis only [22,24] and from cross-sectional and longitudinal analyses performed over the same cohort [20,21] support this hypothesis. A higher proportion of significant findings was, indeed, detected in longitudinal compared to cross-sectional analyses. Another possible argument considers the lack of specificity of the cognitive performance tests used in the literature so far, which only capture neurodevelopment looking at general cognitive abilities or school attainment. This might explain why working with single indexes provided some statistically significant findings in our analysis.

When considering the four statistically significant associations found in the current paper, two of them linked DPs to the VCI in the opposite direction, with the “Seafood” DP being positively related and the “Meat and Potatoes” being inversely related to the VCI. The VCI captures verbal abilities and comprehension, as well as the crystallized intelligence broad factor [57] representing knowledge (knowing what and knowing how) acquired from the environment through acculturation [58]. In childhood, it is parenting and the family environment that have a greater impact on verbal skills, rather than on other performance abilities, more likely linked to the individual’s innate intellectual capacity [59]. On the other hand, child’s dietary habits are influenced by the family environment, especially by maternal dietary habits [24,60]. This common family environment may be responsible for the identified association between the “Meat and Potatoes” or the “Seafood” DP and VCI: while children acquire good and bad dietary habits, they also improve (or not) their verbal skills. 

A finding similar to our “Seafood” DP was reported in the ALSPAC cohort, where the “Health conscious” DP—including fruit and vegetables, pasta, rice, and fish—was positively and significantly related to the verbal IQ and the overall IQ score, as measured with WISC-III [21]. In line with our “Meat and Potatoes” DP, higher scores on the “High fat” DP—based on meat, processed meat, and seafood—were related to lower mean vocabulary test scores in Chinese adolescents [25]. Similarly, in Greek 4-year-old children, the “Snacky” DP—based on potatoes, salty snacks, and sugar products—was inversely associated with verbal ability measured with the McCarthy Scales of Children’s Abilities [18]. The detrimental effect of the “Meat and Potatoes” DP on VCI is generally consistent with previous findings on a Western-style diet. Diets rich in refined carbohydrates and saturated fats negatively impact memory and learning by reducing neuronal proliferation and by up-regulating inflammatory processes that ultimately increase neurodegeneration [61]. Moreover, Jacka and colleagues found that higher scores on a “Western” DP identified in adults correlated to a reduced volume of the hippocampus [62], a brain area thought to be one of the neural bases of crystallized intelligence [63].

The PRI measures both the fluid intelligence broad factor [57], mainly representing deductive and inductive reasoning abilities (i.e., select and process information to find a solution to novel and non-automatically solvable problems) [58], and the visual processing broad factor [57], defined as the ability to cognitively process visual information [58]. In our analysis, children in the highest consumption category of the “Seafood” DP showed a significantly higher mean PRI score. The positive effect of the “Seafood” DP might depend on EPA and DHA intake. Previous studies have linked EPA and DHA to higher brain activity [64,65] and to an increased function in the dorsolateral prefrontal cortex [66], a brain area that is believed to represent one of the neural substrates of fluid intelligence [63,67].

In the absence of other studies linking a fish-based DP to PRI, the “High-energy food pattern”—based on fish snacks together with noodles, eggs, and processed meat—was weakly but significantly related to the perceptual reasoning score measured with the Wechsler Nonverbal Scale of Ability in urban Malay adolescents [19]. Compared to our analysis, the opposite direction of the relationship is likely due to the fried nature of fish snacks, as well as to the additional presence of processed meat [19].

The PSI-related subtests measure the speed of processing broad factor [57], targeting the ability to rapidly perform easy or learned cognitive tasks, in particular when attention is necessary [58]. The PSI is also a measure of the broad factor “Reaction and decision speed” (i.e., the ability to quickly react and make one or several simple decisions in response to simple stimuli) [58]. In our analysis, children in the highest consumption category of the “Dairy Products” DP showed a significantly lower mean PSI score. Our result adds to several others of mixed type [68,69], including recent findings on single cognitive functions and many dairy subgroups or products [70]. We can speculate that our lower mean PSI score depends on the simultaneous presence of high loadings on cholesterol and SFA and low loadings on PUFAs. While PUFAs have been proven to support neural membrane functionality and neurotransmitter action in the frontal lobes (e.g., [71]), a brain area that subsides processing speed [72], they are materially absent in our DP; on the other hand, high intakes of cholesterol and SFA might lead to reduced executing functioning from frontal lobes, thus providing the global detrimental effect of this DP. Similarly, the lack of association of our “Fats” DP with cognitive performance might be due to the simultaneous presence of cholesterol and SFA—showing negative effects—and PUFAs—showing positive effects—on neurodevelopment.

Independently of the cognitive functions considered, a residual role of education and/or income might be at the origin of the identified association between healthier (“Seafood”) or detrimental (“Meat and Potatoes”) DPs and specific cognitive functions [19,73,74] within the so-called family environment. Targeted regression models are needed to consider the more complex patterns including socio-demographic factors, socio-economic factors, diet, and/or cognition, likely within a mediation analysis approach. In addition, in general, a favorable development of the brain may be sustained by healthier DPs in early childhood [21]. These might, in turn, lead to an improved intake of most key nutrients known to be linked to neurodevelopment, such as folate, iodine, iron, and all PUFAs, as well as to improved synergistic effects among them, as captured by DPs [5].

The current analysis has strengths and limitations. Among the strengths, our study shed light on a possible link between higher cognitive functions subsumed by frontal lobe development at 7 years of age [75] and dietary exposure [71]. It also assessed dietary habits through a 3-dDR, which should have provided a more precise quantification of daily, although not necessarily habitual, food intake [76]. The identification of DPs allows to better capture the complexity of dietary exposure and tackles the data dimensionality reduction and the multiple estimation challenges affecting the statistical analysis of many single dietary components. Robust regression models account for the heteroscedasticity of the error term and the presence of outliers; in this way, it reassures that violations of standard assumptions of ordinary least squares methods do not end up in biased results [53]. In addition, although we cannot completely rule out the possibility of residual confounding, we carefully selected a set of ten confounding factors covering different areas of interest (i.e., mother’s, father’s, child’s, and family’s characteristics) and different aspects within the same area (e.g., Raven’s test score, alcohol consumption and folic acid supplementation during pregnancy among mother’s characteristics). The use of several confounding factors, however, reduced the number of children with complete data in the fully adjusted models. In addition, selecting possible confounding factors based on the existing literature led to a high number of potential models inspected compared to the total sample size. Among other possible limitations, NAC-II cohort participants were recruited in one Italian region only, Friuli Venezia Giulia, and with a different aim than assessing the relationship between overall dietary exposure and cognitive performance. However, this different aim likely allowed to track the “Seafood” DP, which was not identified in any of the other papers on *a posteriori* DPs and cognitive performance [18,19,20,21,25]. Although we performed several complementary analyses that reassured on the (internal) reproducibility of the identified DPs [56], we cannot guarantee that the identified DPs are reproducible across different populations [77]. Finally, it is likely that the infant diet plays an important role in later cognitive development [78]. Maternal diet may exert a role as well [60]. We will explore the potential effect of maternal diet, as well as of child’s dietary habits collected at 18 months, on cognitive performance at 18 months, 40 months, and at 7 years in future publications.

## 5. Conclusions

In this population of contemporary Italian 7-year-old children, a concurrent diet based on fish consumption may be associated with better verbal abilities and perceptual reasoning. In addition, diets based on meat and potatoes likely reduced verbal abilities, and those based on dairy products reduced the mean processing speed. Future investigations should integrate cross-sectional and longitudinal analyses within a longitudinal design that uses the same dietary assessment tool, psychometric test, and statistical analysis plan (including control for confounding factors) over multiple administrations to assess the genuine stability of the identified DPs and their relationship with cognitive performance at the available time-points. 

## Figures and Tables

**Table 1:** Socio-demographic, socio-economic, anthropometric, and lifestyle characteristics of enrolled parents and children. Northern Adriatic Cohort II (NAC-II), 2014–2016 (*N* = 379).

Variable	Mean ± SD (Median, 1st–3rd Quartile) or *N* (%)
**Child’s age at neurodevelopmental evaluation (years)**	7 ± 0.05 (7.0, 7.0–7.0)
Missing	0
**Child’s sex**	
Male	195 (51.5)
Female	184 (48.5)
Missing	0
**Child’s body mass index at 7 years**	
Underweight	9 (2.4)
Normal weight	255 (67.3)
Overweight	67 (17.7)
Obese	18 (4.7)
Missing	30 (7.0)
**Child’s extracurricular physical activity at 7 years**	
2 or less days/week	210 (55.4)
3 or more days/week	164 (43.3)
Missing	5 (1.3)
**Child’s birth weight ≥ 4 kg**	
Yes	39 (10.3)
No	336 (88.7)
Missing	4 (1.1)
**Breastfeeding**	
No	22 (5.8)
Yes	338 (89.2)
Missing	19 (5.0)
**Maternal Raven’s test score**	119 ± 11 (125, 114–127)
Missing	0
**Folic acid supplementation before pregnancy**	
No	221 (58.3)
Yes	158 (41.7)
Missing	0
**Alcohol consumption during pregnancy (*n*° of units/week)**	1.6 ± 3.4 (0.3, 0.0–1.5)
Missing	2
**Father’s education**	
Middle school or lower level	109 (28.8)
High school	178 (47.1)
University degree	84 (22.2)
Missing	8 (2.1)
**House property**	
Yes	308 (81.2)
No	67 (17.7)
Missing	4 (1.1)

Abbreviations: SD, standard deviation; Raven’s test score, Raven’s Progressive Matrices Test.

**Table 2:** Distribution of the Full-Scale Intelligence Quotient and the index scores of Wechsler Intelligence Scale for Children–Fourth Edition, in the sample of the children under examination. Northern Adriatic Cohort II (NAC-II), 2014–2016 (*N* = 379).

WISC-IV Index Scores	*N*	Minimum	Mean ± SD	Median (1st–3rd Quartile)	Maximum
Full-Scale Intelligence Quotient (FSIQ)	376	75	109 ± 11	109 (102–116)	141
Verbal Comprehension Index (VCI)	376	76	108 ± 11	108 (100–117)	138
Perceptual Reasoning Index (PRI)	379	82	114 ± 11	113 (106–122)	145
Working Memory Index (WMI)	379	73	98 ± 10	100 (91–106)	133
Processing Speed Index (PSI)	379	62	102 ± 13	103 (91–112)	147

Abbreviations: WISC-IV, Wechsler Intelligence Scale for Children–Fourth Edition.

**Table 3:** Factor loading matrix ^1^, communalities, and explained variances for the five major dietary patterns identified by principal component factor analysis. Northern Adriatic Cohort II (NAC-II), 2014–2016 (*N* = 379).

	Dietary Pattern					
Nutrient	Dairy Products	Plant-Based Foods	Fats	Meat and Potatoes	Seafood	Communality
Protein	0.43	0.00	−0.41	**0.67**	0.15	0.82
Cholesterol	0.41	0.09	−0.48	0.24	0.29	0.54
SFAs	0.46	0.16	**−0.69**	−0.01	−0.02	0.72
MUFAs	0.20	−0.13	**−0.88**	0.14	0.16	0.87
Oleic acid	0.18	−0.15	**−0.87**	0.12	0.15	0.85
Linoleic acid	0.02	−0.16	**−0.61**	0.22	0.10	0.46
Linolenic acid	0.33	−0.19	−0.50	0.12	0.00	0.40
Arachidonic acid	0.06	0.17	−0.26	0.53	0.38	0.52
EPA	−0.02	0.02	0.01	0.11	**0.83**	0.70
DHA	−0.05	−0.04	−0.02	0.01	**0.68**	0.47
Soluble carbohydrates	0.41	−0.48	−0.09	−0.06	−0.14	0.43
Starch	−0.19	−0.33	−0.38	0.42	−0.17	0.49
Fiber	−0.04	**−0.81**	−0.27	0.24	−0.17	0.81
Sodium	−0.01	−0.23	−0.53	0.31	−0.07	0.43
Potassium	0.43	**−0.65**	−0.17	0.46	0.05	0.85
Phosphorus	**0.68**	−0.09	−0.45	0.44	0.09	0.87
Iron	0.17	**−0.61**	−0.27	0.33	0.26	0.65
Zinc	0.37	−0.16	−0.42	**0.60**	0.15	0.73
Selenium	0.24	0.04	0.00	0.29	**0.67**	0.59
Copper	0.43	−0.45	−0.03	0.02	0.44	0.58
Iodine	**0.72**	0.03	−0.03	−0.01	0.25	0.58
Calcium	**0.79**	0.00	−0.32	0.01	−0.12	0.74
Magnesium	**0.75**	−0.35	0.01	0.26	0.08	0.76
Manganese	0.37	−0.59	0.14	0.01	0.09	0.52
Vitamin B1	0.10	−0.44	−0.28	**0.62**	−0.08	0.67
Vitamin B2	**0.67**	−0.26	−0.29	0.37	−0.03	0.73
Niacin	0.09	−0.19	−0.05	**0.82**	0.22	0.76
Pantothenic acid	**0.72**	−0.12	−0.11	0.43	0.22	0.78
Vitamin B6	0.26	−0.49	−0.06	**0.70**	0.10	0.82
Biotin	**0.75**	−0.20	−0.16	0.11	0.18	0.68
Folate	0.16	**−0.69**	−0.25	0.33	−0.08	0.68
Vitamin B12	0.25	−0.07	−0.12	−0.10	0.56	0.41
Retinol	0.35	−0.11	−0.31	−0.22	0.20	0.32
Beta-carotene	−0.07	**−0.64**	−0.10	0.02	−0.01	0.42
Vitamin C	0.15	**−0.75**	0.05	0.05	0.00	0.59
Vitamin D	0.09	0.08	−0.28	0.18	0.56	0.44
Vitamin E ^2^	−0.03	**−0.61**	−0.53	0.01	0.25	0.71
Proportion of explained variance (%)	15.46	13.90	13.40	11.76	8.88	
Cumulative explained variance (%)	15.46	29.35	42.75	54.51	63.39	

^1^ Estimates from a principal component factor analysis carried out on 37 nutrients. For each factor, loadings greater than or equal to 0.60 (in absolute value) indicated important or “dominant nutrients” and are shown in bold typeface. Abbreviations: SFAs, saturated fatty acids; MUFAs, monounsaturated fatty acids; EPA, eicosapentaenoic acid; DHA, docosahexaenoic acid. ^2^ Expressed as alpha-tocopherol equivalents.

**Table 4:** Pearson correlation coefficients ^1^ between continuous factor scores derived from the principal component factor analysis on nutrient intakes and average daily intake (grams) of selected food groups identified on the same participants. Northern Adriatic Cohort II (NAC-II), 2014–2016 (*N* = 379).

Food Group (g/day)	Dairy Products	Plant-Based Foods	Fats	Meat and Potatoes	Seafood
Whole grains and whole bread	−0.11	**0.32**	0.07	0.03	0.00
Refined grains, white bread, and bread substitutes	−0.15	0.11	**0.25**	**0.23**	−0.10
Ready-to-eat meals	−0.17	0.13	0.12	0.06	**−0.20**
Breakfast cereals	0.01	0.14	−0.02	**0.22**	−0.05
Biscuits	0.16	−0.08	0.01	−0.15	−0.05
Milk	**0.56**	−0.18	0.04	0.12	−0.06
Yogurt	**0.39**	−0.06	−0.05	−0.08	−0.14
Milk substitutes	−0.06	**0.23**	0.06	0.03	−0.05
Fat cheese	**0.38**	−0.13	**0.32**	−0.11	−0.09
Low-fat cheese	0.15	0.03	0.17	0.01	−0.04
Eggs	0.18	0.07	0.18	−0.10	0.10
Potatoes	0.04	0.08	−0.04	**0.33**	0.08
Pulses and pulses products	−0.07	**0.22**	0.04	0.09	−0.12
Green leafy vegetables	0.11	**0.34**	0.11	−0.04	0.01
Coloured vegetables	−0.10	**0.38**	0.12	0.00	0.00
Other vegetables	0.08	**0.22**	0.04	0.03	0.01
Citrus fruits	0.02	**0.32**	−0.02	0.03	−0.03
Bananas	**0.20**	**0.36**	**−0.21**	0.03	−0.03
Other fruits	0.15	**0.41**	−0.02	−0.03	−0.02
Fruit juices	0.08	**0.28**	−0.15	−0.04	−0.07
Nuts and seeds	0.13	0.11	0.16	−0.03	−0.04
Fatty fish	0.00	−0.07	0.02	0.03	**0.45**
Lean fish	0.07	0.00	−0.07	0.07	**0.32**
Crustaceans and shellfish	0.06	0.05	−0.08	−0.08	**0.45**
Canned fish	−0.18	−0.01	0.05	−0.03	**0.32**
Processed and ultra-processed meat	−0.04	−0.13	**0.33**	**0.36**	0.05
Poultry	0.10	−0.08	−0.12	**0.44**	0.00
Red meat	0.03	−0.09	0.01	**0.36**	0.09
Spreading fats	0.06	−0.07	0.16	−0.08	0.04
Olive oil and olives	−0.07	**0.20**	**0.42**	−0.08	0.13
Seed oil	−0.06	0.04	0.11	0.09	0.18
Sweet and salty snacks	−0.10	0.05	**0.24**	−0.02	−0.03
Cakes without cream	0.05	0.05	0.17	−0.05	0.13
Spoon desserts and chocolate	0.03	−0.05	**0.23**	−0.07	0.03
Sugar-sweetened beverages	−0.01	−0.10	−0.02	−0.01	−0.06

^1^ Correlations greater or equal to 0.20 (in absolute value) were shown in bold typeface.

**Table 5:** Estimated beta coefficient, standard error (in parenthesis), and corresponding *p*-value derived from robust regression models assessing the relationship between the identified dietary patterns and cognitive performance (WISC-IV), adjusted for selected confounding factors. Northern Adriatic Cohort II (NAC-II), 2014–2016.

	Estimated Beta Coefficient (Standard Error)				
	FSIQ (*N* = 305)	VCI (*N* = 305)	PRI (*N* = 311)	WMI (*N* = 311)	PSI (*N* = 311)
**Intercept**	71.86 (7.36) ***	78.44 (7.43) ***	85.01 (8.10) ***	68.92 (7.15) ***	82.04 (9.87) ***
**Dietary Pattern**					
**Dairy Products**	−0.44 (0.63)	0.13 (0.63)	0.25 (0.69)	0.19 (0.61)	−2.05 (0.84) **
**Plant-based Foods**	−0.02 (0.66)	0.30 (0.67)	0.00 (0.72)	0.57 (0.64)	−1.12 (0.88)
**Fats**	−0.38 (0.61)	−0.98 (0.62)	0.13 (0.68)	−0.45 (0.60)	0.42 (0.83)
**Meat and Potatoes**	−0.88 (0.65)	−1.28 (0.66) *	−1.07 (0.72)	−0.22 (0.63)	0.51 (0.87)
**Seafood**	0.90 (0.63)	1.24 (0.64) *	1.35 (0.70) *	−0.45 (0.62)	−0.30 (0.85)
**Child’s characteristic**					
**Sex**	−1.39 (1.24)	−1.30 (1.26)	−4.26 (1.36) ***	−0.96 (1.20)	3.26 (1.66) **
**Body mass index**					
Obese	−4.11 (2.71)	−4.24 (2.74)	0.76 (3.00)	−2.72 (2.65)	−5.26 (3.66)
Overweight	−0.67 (1.58)	0.72 (1.59)	−0.74 (1.73)	−2.50 (1.53)	1.01 (2.11)
Underweight	−3.60 (3.80)	−0.37 (3.84)	−3.53 (4.20)	−2.89 (3.71)	−1.25 (5.12)
**Child’s extracurricular physical activity at 7 years of age**	1.27 (1.27)	0.84 (1.28)	−0.42 (1.40)	1.08 (1.24)	2.42 (1.71)
**Child’s birth weight ≥ 4 kg**	−0.76 (2.03)	−1.08 (2.05)	1.77 (2.21)	−0.68 (1.95)	−4.38 (2.70)
**Breastfeeding**	3.39 (2.68)	3.39 (2.71)	0.69 (2.97)	3.77 (2.62)	1.53 (3.62)
**Mother’s characteristic**					
**Raven’s test score**	0.25 (0.06) ***	0.20 (0.06) ***	0.22 (0.06) ***	0.18 (0.06) ***	0.12 (0.08)
**Folic acid supplementation before pregnancy**	3.09 (1.24) **	2.51 (1.25) **	2.04 (1.37)	2.00 (1.20) *	1.94 (1.66)
**Alcohol consumption during pregnancy (n° of units/week)**	−0.12 (0.23)	−0.72 (0.23) ***	0.18 (0.25)	−0.01 (0.22)	0.32 (0.31)
**Father’s characteristic**					
**Father’s education**					
High school	3.73 (1.43) ***	3.20 (1.45) **	3.05 (1.57) *	3.43 (1.39) **	2.00 (1.92)
University degree	5.93 (1.80) ***	4.15 (1.82) **	4.52 (1.99) **	6.65 (1.76) ***	2.41 (2.43)
**Family’s characteristic**					
**House property**	2.61 (1.72)	2.55 (1.74)	0.16 (1.90)	2.03 (1.68)	2.22 (2.32)

Estimates of beta coefficients were derived from robust regression models, including factor scores from each of the five dietary patterns simultaneously and several confounding factors. We referred to Supplementary Table S5 for the distribution of each factor score in the examined sample. The reference categories for each confounder included in the models were as follows: child’s sex: male; child’s body mass index at 7 years of age: normal weight status; child’s extracurricular physical activity at 7 years: 2 or less days/week; child’s birth weight ≥ 4 kg: no; breastfeeding: no; folic acid supplementation before pregnancy: no; father’s education: middle school or lower level; house property: no. Significance codes for the *p*-values: ‘***’ < 0.001 ‘**’ < 0.01 ‘*’ < 0.05. Abbreviations: FSIQ, full-scale intelligence quotient; VCI, verbal comprehension index; PRI, perceptual reasoning index; WMI, working memory index; PSI, processing speed index.

## Data Availability

The data described in the manuscript, in the code book, and in the analytical code are available upon motivated request.

## Supplementary Materials

The following supporting information can be downloaded at: https://www.mdpi.com/article/10.3390/nu14194168/s1, Table S1: Detailed description of content and daily intakes (g/day) of each food group. Table S2: Lifestyle and socio-environmental characteristics of enrolled children. Northern Adriatic Cohort II (NAC-II), 2014–2016 (*N* = 379); Table S3: Factorability of the correlation matrix of the original nutrients: individual and overall measures of sampling adequacy and Bartlett’s test of sphericity; Table S4: Standardized Cronbach’s coefficient alpha for each factor and standardized Cronbach’s coefficient alpha when-item-deleted for each factor and nutrient.; Table S5: Distribution of factor scores in the sample of enrolled children. Northern Adriatic Cohort II (NAC-II), 2014–2016 (*N* = 379); Table S6: Estimated beta coefficient, standard error (in parenthesis), and corresponding *p*-value derived from robust regression models assessing the relationship between the identified dietary patterns and cognitive performance (WISC-IV), adjusted for selected confounding factors including variables on social environment at home. Northern Adriatic Cohort II (NAC-II), 2014–2016; Table S7: Estimated beta coefficient, standard error (in parenthesis), and corresponding *p*-value derived from robust regression models assessing the relationship between the identified dietary patterns and cognitive performance (WISC-IV), adjusted for selected confounding factors including child’s device use during weekdays and weekend. Northern Adriatic Cohort II (NAC-II), 2014–2016; Table S8: Estimated beta coefficient, standard error (in parenthesis), and corresponding *p*-value derived from robust regression models assessing the relationship between the identified dietary patterns and cognitive performance (WISC-IV), adjusted for selected confounding factors including child’s TV use during weekdays and weekend. Northern Adriatic Cohort II (NAC-II), 2014–2016; Table S9: Estimated beta coefficient, standard error (in parenthesis), and corresponding *p*-value derived from robust regression models assessing the relationship between the identified dietary patterns and cognitive performance (WISC-IV), adjusted for selected confounding factors and restricted to full-term children. Northern Adriatic Cohort II (NAC-II), 2014–2016.
