# Supplementary material for: Are Dietary Patterns Related to Cognitive Performance in 7-Year-Old Children? Evidence from a Birth Cohort in Friuli Venezia Giulia, Italy
Source: Nutrients. 2022 Oct 7;14(19):4168. doi: 10.3390/nu14194168 (PMC9571625; doi:10.3390/nu14194168)
Supplement: Supplementary file 1 [file nutrients-14-04168-s001.zip › nutrients-1943036-supplementary.pdf]

Supplementary Materials

Are dietary patterns related to cognitive performance in 7-year-old children? Evidence from a birth cohort in Friuli Venezia Giulia, Italy.

Michela Marinoni, Elisa Giordani, Cedric Mosconi, Valentina Rosolen, Federica Concina, Federica Fiori, Claudia Carletti, Alessandra Knowles, Paola Pani, Maura Bin, Luca Ronfani, Monica Ferraroni, Fabio Barbone, Maria Parpinel, and Valeria Edefonti

Supplementary Table S1. Detailed description of content and daily intakes (g/day) of each food group.

Food Group	Description (main food items)	Median (1st–3rd quantile)	NCs (%)
Whole grains and whole bread	Whole pasta, rice, and other cereals, whole grain flours and bread	0.0 (0.0–0.0)	85.5
Refined grains, white bread and bread substitutes	Refined pasta, rice, and other polished cereals, refined grain flours, bread, crackers, breadsticks	102.8 (74.4–128.5)	0.0
Ready-to-eat meals	Frozen pizza, tortellini, lasagna, gnocchi, soups	23.3 (0.0–55.9)	43.3
Breakfast cereals	Corn flakes, muesli, puffed rice, oat flakes	0.0 (0.0–8.3)	65.7
Biscuits	Biscuits, wafers, chocolate cookies, butter cookies	17.3 (3.0–33.5)	22.4
Milk	Whole milk, semi-skimmed milk	140.5 (52.4–212.0)	5.5
Yogurt	Unflavored/flavoured yogurt	38.3 (0.0–83.3)	42.7
Milk substitutes	Soy and rice beverages	0.0 (0.0–0.0)	96.3
Fat cheese	*Parmigiano* cheese, *latteria* cheese, processed cheese	12.1 (3.9–24.3)	9.2
Low-fat cheese	Ricotta, mozzarella, feta cheese	0.0 (0.0–11.5)	63.1
Eggs	Eggs	5.2 (0.8–17.0)	21.6
Potatoes	Potatoes, mashed potatoes	21.3 (3.7–41.1)	23.5
Pulses and pulses products	Lentils, peas, chickpeas, beans and derivatives	2.5 (0.0–17.5)	44.9
Green leafy vegetables	Lettuce, spinach, chard	8.1 (0.0–25.0)	35.9
Coloured vegetables	Tomatoes, carrots, zucchini, aubergines, peppers	36.0 (16.8–70.6)	4.2
Other vegetables	Celery, cucumbers, onions, radicchio, green beans	13.2 (3.5–32.7)	5.8
Citrus fruits	Whole and squeezed oranges, grapefruit, tangerines, and lemons	24.1 (0.0–24.1)	58.8
Bananas	Bananas	0.0 (0.0–43.39	63.1
Other fruits	Apples, pears, peaches, kiwi, watermelon	75.0 (27.5–126.7)	19.0
Fruit juices	Sweetened and non-sweetened juices	0.0 (0.0–101.7)	50.4
Nuts and seeds	Nuts, peanuts, flaxseed	0.0 (0.0–0.0)	77.6
Fatty fish	Sardines, mackerel, salmon	0.0 (0.0–0.0)	93.4
Lean fish	Cod, sole, trout	0.0 (0.0–0.0)	78.6
Crustaceans and shellfish	Mussels, clams, squid, cuttlefish	0.0 (0.0–0.0)	88.7
Canned fish	Tuna, mackerel, sardines, smoked salmon	0.0 (0.0–2.7)	73.4
Processed and ultra-processed meat	Bacon, sausage, ham, salami, nuggets	20.0 (10.0–39.1)	8.2

Poultry	Chicken, turkey	20.0 (0.0–40.0)	42.5
Red meat	Pork, beef, horse, lamb, duck	20.3 (2.6–41.0)	20.1
Spreading fats	Butter and cream	2.2 (0.1–6.7)	20.1
Olive oil and olives	Extra-vergin olive oil, olive oil, olives and olive patè	6.5 (4.0–10.5)	0.5
Seed oil	Peanut, corn, and sunflower	0.0 (0.0–2.9)	50.4
Sweet and salty snacks	Brioches, pastries, cakes with cream, popcorn, chips	20.0 (8.7–35.8)	9.5
Cakes without cream	Tart, sponge cake, chocolate cake	0.0 (0.0–0.0)	82.1
Spoon desserts and chocolate	Ice-cream, puddings, cocoa powder, chocolate	22.0 (7.1–40.6)	8.7
Sugar-sweetened beverages	Cola, ice tea, orange soda	0.0 (0.0–0.0)	76.3

Abbreviations: NCs, non-consumers.

Supplementary Table S2. Lifestyle and socio-environmental characteristics of enrolled children. Northern Adriatic Cohort II (NAC-II), 2014–2016 (N = 379).

Variable	Mean±SD (median, 1st–3rd quartile) or N (%)
**Use of any device during weekdays**	
Never	80 (21.1)
< 1 hour/day	171 (45.1)
≥ 1 hour/day	68 (18.0)
Missing	60 (15.8)
**Use of any device during weekend**	
Never	41 (10.8)
< 1 hour/day	152 (40.1)
≥ 1 hour/day	126 (33.2)
Missing	60 (15.8)
**Watching TV during weekdays**	
< 1 hour/day	119 (31.4)
≥ 1 hour/day	254 (67.0)
Missing	6 (1.6)
**Watching TV during weekend**	
< 1 hour/day	56 (14.8)
≥ 1 hour/day	318 (84.0)
Missing	5 (1.3)
**Child's age at AIRE assessment (months)**	31.6±2.9 (32.0, 30.0–34.0)
Missing	48
**Communication and affective interaction between parents and child**	18.4±1.7 (19.0, 18.0–20.0)
Missing	48
**Promotion of child autonomy**	18.8±1.4 (19.0, 18.0–20.0)
Missing	48
**Respect for the child and implementation of rules**	16.5±2.3 (17.0, 15.0–18.0)
Missing	48
**Emotional atmosphere**	17.2±2.3 (18.0, 16.0–19.0)
Missing	48
**Pre-term births**	
≥ 37 weeks	368 (97.1)
<37 weeks	5 (1.3)
Missing	6 (1.6)

The AIRE instrument is defined by 4 scores: A) affective interaction and communication between parents and child (i.e. mother spontaneously vocalizes to the child; the mother caresses or kisses the child); I) promotion of child autonomy (i.e. there are toys and activities for intellectual development, mother encourage the child to eat alone), R) respect for the child and implementation of rules (i.e. there is good compliance between parents about the home rules; caregivers do not shout at the child during the visit); E) emotional atmosphere (i.e. father provides some care-giving every day; family visits or receives visits from relatives). The range of possible scores of the AIRE is 0–20 for each subscale. Abbreviations: SD, standard deviation.

Supplementary Table S3. Factorability of the correlation matrix of the original nutrients: individual and overall measures of sampling adequacy and Bartlett's test of sphericity.

**Bartlett's Test of Sphericity:** p-value < 0.001	
**Overall Measure of Sampling Adequacy (Kaiser–Meyer–Olkin statistic)[1]:** 0.84	
**Individual Measures of Sampling Adequacy[1]:**	
<0.60	DHA, vitamin B12
0.60-0.69	EPA, retinol
0.70-0.79	Soluble carbohydrates, beta-carotene, niacin, vitamin D, selenium
0.80-0.89	MUFAs, oleic acid, calcium, manganese, sodium, linolenic acid, starch, linoleic acid, proteins, vitamin E, iodine, vitamin C, SFAs, vitamin B2, pantothenic acid, fiber, iron, arachidonic acid, cholesterol, folate, biotin, copper, vitamin B6
≥0.90	Phosphorus, vitamin B1, potassium, zinc, magnesium

[1] Overall and individual measures of sampling adequacy range between 0 and 1, with values > 0.60 indicating a satisfactory size. Abbreviations: SFAs, saturated fatty acids; MUFAs, monounsaturated fatty acids; EPA, eicosapentaenoic acid; DHA, docosahexaenoic acid.

Supplementary Table S4. Standardized Cronbach's coefficient alpha[1,2] for each factor and standardized Cronbach's coefficient alpha when-item-deleted[a] for each factor and nutrient.

Nutrient	Dietary Pattern				
	Dairy Products	Plant-based Foods	Fats	Meat and Potatoes	Seafood
		Standardized alpha			
Standardized alpha	0.92	0.90	0.91	0.91	0.75
		Standardized alpha when-item-deleted			
Protein	0.91		0.90	0.89	
Cholesterol	0.92		0.90		
SFAs	0.92		0.90		
MUFAs			0.89		
Oleic acid			0.89		
Linoleic acid			0.90		
Linolenic acid			0.90		
Arachidonic acid				0.91	
EPA					0.64
DHA					0.72
Soluble carbohydrates	0.92	0.90			
Starch				0.92	
Fiber		0.89			
Sodium			0.91		
Potassium	0.91	0.88		0.90	
Phosphorus	0.91		0.90	0.90	
Iron		0.89			
Zinc			0.90	0.89	
Selenium					0.67
Copper	0.92	0.90			0.73
Iodine	0.92				
Calcium	0.91				
Magnesium	0.91				
Manganese		0.90			
Vitamin B1		0.90		0.90	
Vitamin B2	0.91				
Niacin				0.90	
Pantothenic acid	0.91			0.90	
Vitamin B6		0.89		0.89	
Biotin	0.91				
Folate		0.89			
Vitamin B12					0.74
Retinol					
Beta-carotene		0.90			
Vitamin C		0.89			
Vitamin D					
Vitamin E [3]		0.90	0.91		

[1] Cronbach's coefficient alphas generally range between 0 and 1.
[2] We evaluated Cronbach's coefficient alphas considering only those nutrients that load more than 0.40 (in absolute value) on any factor. For this reason, some cells in the table were empty.
[3] Expressed as alpha-tocopherol equivalents.

Supplementary Table S5. Distribution of factor scores in the sample of enrolled children. Northern Adriatic Cohort II (NAC-II), 2014–2016 (N = 379).

Factor score	N	Minimum	Mean±SD	Median (1st–3rd quartile)	Maximum
Diary Products	379	-2.25	0.00±1.00	-0.06 (-0.73-0.65)	3.86
Plant-based Foods[1]	379	-2.09	0.00±1.00	-0.16 (-0.67-0.49)	4.86
Fats[1]	379	-2.83	0.00±1.00	-0.10 (-0.65–0.53)	3.98
Meat and Potatoes	379	-4.20	0.00±1.00	-0.09 (-0.68–0.60)	3.22
Seafood	379	-1.88	0.00±1.00	-0.29 (-0.62–0.42)	5.38

[1] We reversed the sign of factor scores for the "Plant-based Foods" and the "Fats" dietary patterns (showing most nutrients with negative factor loadings), to make them comparable with scores from the other dietary patterns in the regression models.

Supplementary Table S6. Estimated beta coefficient, standard error (in parenthesis), and corresponding p-value derived from robust regression models assessing the relationship between the identified dietary patterns and cognitive performance (WISC-IV), adjusted for selected confounding factors including variables on social environment at home. Northern Adriatic Cohort II (NAC-II), 2014–2016.

	Estimated Beta Coefficient (Standard Error)				
	**FSIQ (N=269)**	**VCI (N=269)**	**PRI (N=275)**	**WMI (N=275)**	**PSI (N=275)**
**Intercept**	49.31 (13.18) ***	42.8 (13.1) ***	68.04 (15.23) ***	43.71 (12.88) ***	103.71 (17.86) ***
**Dietary Pattern**					
Dairy Products	-0.19 (0.66)	0.18 (0.66)	0.49 (0.76)	0.31 (0.64)	-1.44 (0.89)
Plant-based foods	-0.28 (0.70)	0.40 (0.70)	-0.07 (0.8)	0.20 (0.68)	-1.24 (0.94)
Fats	-0.12 (0.66)	-0.46 (0.66)	0.20 (0.77)	-0.22 (0.65)	0.51 (0.90)
Meat and Potatoes	-0.95 (0.69)	-1.56 (0.69) **	-1.02 (0.8)	-0.44 (0.67)	0.92 (0.94)
Seafood	1.06 (0.66)	0.98 (0.66)	1.64 (0.76) **	-0.24 (0.65)	0.06 (0.89)
**Child's characteristic**					
**Sex**	-2.64 (1.31) **	-3.1 (1.30) **	-4.95 (1.5) ***	-2.32 (1.27) *	3.25 (1.76) *
**Body mass index**					
Obese	-3.95 (2.84)	-7.41 (2.83) ***	2.68 (3.29)	-3.23 (2.78)	-4.55 (3.86)
Overweight	-0.84 (1.68)	-0.35 (1.67)	-1.27 (1.93)	-2.53 (1.63)	2.16 (2.26)
Underweight	-3.47 (4.05)	-2.26 (4.02)	-5.98 (4.69)	-2.33 (3.96)	3.47 (5.50)
**Child's extracurricular physical activity at 7 years**	1.10 (1.36)	0.39 (1.35)	-1.10 (1.56)	0.55 (1.32)	3.29 (1.84) *
**Child's birth weight ≥ 4 kg**	-0.39 (2.05)	-0.24 (2.04)	2.40 (2.34)	-0.59 (1.98)	-4.30 (2.74)
**Breastfeeding**	2.08 (2.94)	5.2 (2.92) *	0.58 (3.41)	2.05 (2.88)	-2.15 (3.99)
**Mother's characteristic**					
**Raven's test score**	0.22 (0.06) ***	0.15 (0.06) **	0.24 (0.07) ***	0.17 (0.06) ***	0.07 (0.08)

**Folic acid supplementation before pregnancy**	3.12 (1.29) **	2.45 (1.28) *	2.69 (1.49) *	1.92 (1.26)	1.01 (1.74)
**Alcohol consumption during pregnancy (n° of units/week)**	-0.15 (0.23)	-0.79 (0.23) ***	0.11 (0.27)	0.04 (0.23)	0.37 (0.31)
**Father's characteristic**					
**Father's education**					
High school	2.90 (1.55) *	3.08 (1.54) **	1.96 (1.78)	2.46 (1.51)	2.22 (2.09)
University degree	5.06 (1.89) ***	3.92 (1.88) **	4.40 (2.18) **	5.67 (1.84) ***	1.10 (2.56)
**Family's characteristic**					
**House property**	2.09 (1.94)	1.11 (1.92)	0.35 (2.24)	1.34 (1.89)	3.17 (2.62)
**AIRE scores**					
Child's age at AIRE assessment (months)	0.21 (0.23)	0.15 (0.23)	-0.10 (0.26)	0.23 (0.22)	0.26 (0.31)
Communication and affective interaction between parents and child	-0.51 (0.47)	-0.12 (0.47)	0.34 (0.54)	0.04 (0.46)	-2.00 (0.64) ***
Promotion of child autonomy	0.88 (0.55)	1.39 (0.55) **	0.52 (0.64)	0.74 (0.54)	-0.32 (0.75)
Respect for the child and implementation of rules	0.22 (0.36)	0.06 (0.36)	0.12 (0.41)	0.15 (0.35)	0.46 (0.49)
Emotional atmosphere	0.62 (0.35) *	0.70 (0.35) **	0.07 (0.40)	0.34 (0.34)	0.84 (0.48) *

Estimates of beta coefficients were derived from robust regression models including the five dietary patterns simultaneously and several confounding factors. The reference categories for each confounder included in the models were as follows: Child's sex: male; Child's body mass index at 7 years: normal weight status; Child's extracurricular physical activity at 7 years: 2 or less days/week; Child's birth weight ≥ 4 kg: no; Breastfeeding: no; Folic acid supplementation before pregnancy: no; Father's education: middle school or lower level; House property: no. Significance codes for the p-values: '***' <0.001 '**' <0.01 '*' <0.05. Abbreviations: WISC-IV, Wechsler Intelligence Scale for Children 4th Edition; FSIQ, Full Scale Intelligence Quotient; VCI, Verbal Comprehension Index; PRI, Perceptual Reasoning Index; WMI, Working Memory Index; PSI, Processing Speed Index.

Supplementary Table S7. Estimated beta coefficient, standard error (in parenthesis), and corresponding p-value derived from robust regression models assessing the relationship between the identified dietary patterns and cognitive performance (WISC-IV), adjusted for selected confounding factors including child's device use during weekdays and weekend. Northern Adriatic Cohort II (NAC-II), 2014–2016.

	Estimated Beta Coefficient (Standard Error)				
	**FSIQ (N=264)**	**VCI (N=264)**	**PRI (N=268)**	**WMI (N=268)**	**PSI (N=268)**
**Intercept**	75.26 (8.00) ***	77.56 (8.08) ***	90.26 (8.63) ***	72.43 (7.95) ***	83.13 (10.77) ***
**Dietary Pattern**					
Dairy Products	0.07 (0.70)	0.55 (0.70)	0.66 (0.75)	0.2 (0.69)	-1.55 (0.93) *
Plant-based foods	0.68 (0.78)	1.15 (0.79)	0.27 (0.84)	1.16 (0.78)	-0.91 (1.05)
Fats	-0.63 (0.69)	-1.2 (0.7) *	-0.03 (0.75)	-0.38 (0.69)	0.14 (0.93)
Meat and Potatoes	-1.26 (0.73) *	-1.86 (0.74) **	-1.59 (0.79) **	-0.59 (0.72)	1.05 (0.98)
Seafood	0.5 (0.69)	1.37 (0.7) *	0.95 (0.75)	-1.04 (0.69)	-0.72 (0.93)
**Child's characteristic**					
**Sex**	-2.11 (1.41)	-2.03 (1.42)	-3.9 (1.51) **	-1.53 (1.39)	1.89 (1.89)
**Body mass index**					
Obese	-4.35 (2.86)	-4.32 (2.88)	0.56 (3.08)	-3.24 (2.84)	-5.14 (3.84)
Overweight	-1.35 (1.71)	0.3 (1.73)	-2.35 (1.85)	-2.81 (1.7) *	1.04 (2.3)
Underweight	-1.54 (4.13)	-0.87 (4.18)	-1.04 (4.47)	-1.93 (4.11)	3.03 (5.57)
**Child's extracurricular physical activity at 7 years**	1.22 (1.38)	0.69 (1.4)	-0.25 (1.49)	1.34 (1.37)	2.28 (1.86)
**Child's birth weight ≥ 4 kg**	-0.7 (2.22)	-1.08 (2.24)	0.89 (2.39)	0.19 (2.2)	-4.75 (2.99)
**Breastfeeding**	3.17 (2.91)	3.51 (2.94)	-0.49 (3.14)	2.98 (2.89)	2.33 (3.92)
**Use of any device during weekdays**					
< 1 hour/day	1.64 (1.82)	1.44 (1.83)	1.66 (1.96)	-0.52 (1.8)	1.3 (2.44)

≥ 1 hour/day	-2.26 (2.46)	0.89 (2.48)	-4.7 (2.65) *	-1.27 (2.44)	-3.57 (3.31)
**Use of any device during weekend**					
< 1 hour/day	3.45 (2.22)	2.08 (2.24)	4.18 (2.4) *	3.41 (2.21)	0.86 (2.99)
≥ 1 hour/day	2.72 (2.48)	0.49 (2.51)	4.44 (2.68) *	1.1 (2.47)	3.79 (3.35)
**Mother's characteristic**					
**Raven's test score**	0.2 (0.06) ***	0.2 (0.06) ***	0.15 (0.07) **	0.16 (0.06) **	0.09 (0.08)
**Folic acid supplementation before pregnancy**	2.59 (1.35) *	2.19 (1.37)	2.03 (1.46)	1.85 (1.34)	1.61 (1.82)
**Alcohol consumption during pregnancy (n° of units/week)**	0.01 (0.26)	-0.69 (0.26) ***	0.31 (0.28)	0.01 (0.26)	0.65 (0.35) *
**Father's characteristic**					
**Father's education**					
High school	4.35 (1.56) ***	3.51 (1.57) **	3.63 (1.68) **	2.97 (1.54) *	2.64 (2.09)
University degree	6.02 (1.94) ***	3.97 (1.96) **	4.59 (2.1) **	6.33 (1.93) ***	3.45 (2.62)
**Family's characteristic**					
**House property**	2.11 (1.82)	2.14 (1.84)	0.53 (1.96)	1.47 (1.81)	1.6 (2.45)

Estimates of beta coefficients were derived from robust regression models including the five dietary patterns simultaneously and several confounding factors. The reference categories for each confounder included in the models were as follows: Child's sex: male; Child's body mass index at 7 years: normal weight status; Child's extracurricular physical activity at 7 years: 2 or less days/week; Child's birth weight ≥ 4 kg: no; Breastfeeding: no; Folic acid supplementation before pregnancy: no; Father's education: middle school or lower level; House property: no; Use of any device during weekdays: never; Use of any device during weekend: never. Significance codes for the p-values: '***' <0.001 '**' <0.01 '*' <0.05.

Supplementary Table S8. Estimated beta coefficient, standard error (in parenthesis), and corresponding p-value derived from robust regression models assessing the relationship between the identified dietary patterns and cognitive performance (WISC-IV), adjusted for selected confounding factors including child's TV use during weekdays and weekend. Northern Adriatic Cohort II (NAC-II), 2014–2016.

	**Estimated Beta Coefficient (Standard Error)**				
	**FSIQ (N=303)**	**VCI (N=303)**	**PRI (N=309)**	**WMI (N=309)**	**PSI (N=309)**
**Intercept**	72.27 (7.50) ***	77.61 (7.57) ***	84.65 (8.33) ***	71.57 (7.29) ***	83.32 (10.07) ***
**Dietary Pattern**					
Dairy Products	-0.49 (0.63)	0.08 (0.64)	0.23 (0.7)	0.26 (0.61)	-2.11 (0.84) **
Plant-based foods	-0.02 (0.66)	0.35 (0.67)	0.04 (0.73)	0.49 (0.64)	-1.22 (0.88)
Fats	-0.38 (0.62)	-1.03 (0.63) *	0.12 (0.69)	-0.38 (0.6)	0.51 (0.83)
Meat and Potatoes	-0.9 (0.67)	-1.23 (0.67) *	-1.05 (0.73)	-0.02 (0.64)	0.31 (0.89)
Seafood	0.92 (0.63)	1.24 (0.64) *	1.33 (0.71) *	-0.42 (0.62)	-0.24 (0.85)
**Child's characteristic**					
**Sex**	-1.44 (1.25)	-1.5 (1.26)	-4.42 (1.38) ***	-0.68 (1.21)	3.38 (1.67) **
**Body mass index**					
Obese	-3.95 (2.72)	-4.43 (2.75)	0.67 (3.03)	-2.5 (2.66)	-4.99 (3.67)
Overweight	-0.56 (1.58)	0.59 (1.6)	-0.8 (1.75)	-2.35 (1.53)	1.25 (2.11)
Underweight	-3.43 (3.81)	-0.58 (3.85)	-3.62 (4.25)	-2.48 (3.72)	-0.76 (5.14)
**Child's extracurricular physical activity at 7 years**	1.33 (1.28)	0.8 (1.29)	-0.42 (1.42)	0.91 (1.24)	2.72 (1.72)
**Child's birth weight ≥ 4 kg**	-0.94 (2.04)	-1.37 (2.06)	1.65 (2.24)	-0.87 (1.96)	-4.27 (2.7)
**Breastfeeding**	3.56 (2.69)	3.63 (2.71)	0.73 (2.99)	3.65 (2.62)	1.62 (3.62)
**Watching television**					
During weekdays (≥ 1 hour/day)	0.18 (1.49)	1.22 (1.50)	0.12 (1.65)	0.71 (1.44)	-1.69 (1.99)

During weekend (≥ 1 hour/day)	-1.58 (1.99)	-1.07 (2.01)	-0.10 (2.20)	-3.95 (1.92) **	0.05 (2.66)
**Mother's characteristic**					
**Raven's test score**	0.25 (0.06) ***	0.21 (0.06) ***	0.23 (0.07) ***	0.18 (0.06) ***	0.12 (0.08)
**Folic acid supplementation before pregnancy**	3.31 (1.25) ***	2.53 (1.27) **	2 (1.39)	2.4 (1.22) **	2.23 (1.68)
**Alcohol consumption during pregnancy (n° of units/week)**	-0.11 (0.23)	-0.73 (0.23) ***	0.17 (0.25)	0.05 (0.22)	0.33 (0.31)
**Father's characteristic**					
**Father's education**					
High school	3.78 (1.44) ***	3.4 (1.45) **	3.14 (1.59) **	3.16 (1.39) **	1.91 (1.92)
University degree	5.89 (1.82) ***	4.39 (1.83) **	4.73 (2.02) **	6.46 (1.77) ***	1.86 (2.44)
**Family's characteristic**					
**House property**	2.54 (1.72)	2.38 (1.74)	0.06 (1.92)	2.11 (1.68)	2.28 (2.32)

Estimates of beta coefficients were derived from robust regression models including the five dietary patterns simultaneously and several confounding factors. The reference categories for each confounder included in the models were as follows: Child's sex: Male; Child's body mass index at 7 years: Normal weight; Child's extracurricular physical activity at 7 years: 2 or less days/week; Child's birth weight ≥ 4 kg: no; Breastfeeding: no; Folic acid supplementation before pregnancy: no; Father's education: middle school or lower level; House property: no; Watching television during weekdays: less than 1 hour/day; Watching television during weekend: less than 1 hour/day. Significance codes for the p-values: '***' <0.001 '**' <0.01 '*' <0.05.

Supplementary Table S9. Estimated beta coefficient, standard error (in parenthesis), and corresponding p-value derived from robust regression models assessing the relationship between the identified dietary patterns and cognitive performance (WISC-IV), adjusted for selected confounding factors and restricted to full-term children. Northern Adriatic Cohort II (NAC-II), 2014–2016.

	Estimated Beta Coefficient (Standard Error)				
	**FSIQ (N=305)**	**VCI (N=305)**	**PRI (N=311)**	**WMI (N=311)**	**PSI (N=311)**
**Intercept**	72.66 (7.35) ***	78.53 (7.48) ***	86.95 (8.09) ***	69.31 (7.17) ***	81.96 (9.9) ***
**Dietary Pattern**					
Dairy Products	-0.45 (0.63)	0.18 (0.64)	0.24 (0.68)	0.17 (0.61)	-2.02 (0.84) **
Plant-based foods	0.18 (0.67)	0.3 (0.68)	0.3 (0.73)	0.74 (0.65)	-1.01 (0.89)
Fats	-0.5 (0.61)	-1.07 (0.63) *	0.09 (0.68)	-0.54 (0.6)	0.23 (0.83)
Meat and Potatoes	-0.85 (0.65)	-1.19 (0.66) *	-1.06 (0.71)	-0.2 (0.63)	0.65 (0.87)
Seafood	0.95 (0.63)	1.33 (0.64) **	1.47 (0.7) **	-0.45 (0.62)	-0.28 (0.85)
**Child's characteristic**					
**Sex**	-1.65 (1.24)	-1.48 (1.27)	-4.69 (1.36) ***	-1.1 (1.21)	3.16 (1.67) *
**Body mass index**					
Obese	-5.02 (2.77) *	-4.41 (2.82)	-0.43 (3.06)	-3.34 (2.72)	-5.76 (3.75)
Overweight	-0.89 (1.58)	0.57 (1.61)	-1.09 (1.73)	-2.78 (1.53) *	0.88 (2.12)
Underweight	-3.64 (3.78)	-0.3 (3.84)	-3.61 (4.17)	-3.01 (3.7)	-1.24 (5.11)
**Child's extracurricular physical activity at 7 years**	1.09 (1.28)	0.6 (1.3)	-0.33 (1.41)	0.97 (1.25)	2.03 (1.72)
**Child's birth weight ≥ 4 kg**	-0.56 (2.02)	-0.94 (2.06)	1.89 (2.2)	-0.52 (1.95)	-4.13 (2.69)
**Breastfeeding**	3.2 (2.67)	3.27 (2.72)	0.52 (2.95)	3.61 (2.61)	1.19 (3.61)
**Mother's characteristic**					
**Raven's test score**	0.25 (0.06) ***	0.2 (0.06) ***	0.21 (0.06) ***	0.18 (0.06) ***	0.12 (0.08)

**Folic acid supplementation before pregnancy**	3.3 (1.25) ***	2.35 (1.27) *	2.22 (1.37)	2.34 (1.22) *	2.14 (1.68)
**Alcohol consumption during pregnancy (n° of units/week)**	-0.17 (0.23)	-0.76 (0.23) ***	0.13 (0.25)	-0.04 (0.22)	0.29 (0.31)
**Father's characteristic**					
**Father's education**					
High school	3.36 (1.43) **	3.14 (1.46) **	2.67 (1.57) *	3.08 (1.39) **	1.6 (1.93)
University degree	5.93 (1.8) ***	4.3 (1.83) **	4.45 (1.98) **	6.54 (1.75) ***	2.42 (2.42)
**Family's characteristic**					
**House property**	2.68 (1.74)	2.18 (1.77)	0.23 (1.92)	2.33 (1.7)	2.24 (2.35)

Estimates of beta coefficients were derived from robust regression models including the five dietary patterns simultaneously and several confounding factors, restricted to the full-term children. The reference categories for each confounder included in the models were as follows: Child's sex: male; Child's body mass index at 7 years: normal weight; Child's extracurricular physical activity at 7 years: 2 or less days/week; Child's birth weight ≥ 4 kg: no; Breastfeeding: no; Folic acid supplementation before pregnancy: no; Father's education: middle school or lower level; House property: no. Significance codes for the p-values: '***' <0.001 '**' <0.01 '*' <0.05.
